# Enhancing tomato fruit antioxidant potential through hydrogen nanobubble irrigation

**DOI:** 10.1093/hr/uhae111

**Published:** 2024-04-16

**Authors:** Jing He, Yunpeng Zhou, Christoph-Martin Geilfus, Jiankang Cao, Daqi Fu, Shahar Baram, Yanzheng Liu, Yunkai Li

**Affiliations:** State Key Laboratory of Efficient Utilization of Agricultural Water Resources, China Agricultural University, Beijing 100083, China; Engineering Research Center for Agricultural Water-Saving and Water Resources, Ministry of Education, China Agricultural University, Beijing 100083, China; State Key Laboratory of Efficient Utilization of Agricultural Water Resources, China Agricultural University, Beijing 100083, China; Engineering Research Center for Agricultural Water-Saving and Water Resources, Ministry of Education, China Agricultural University, Beijing 100083, China; Department of Soil Science & Plant Nutrition, Hochschule Geisenheim University, Hessen 65366, Germany; College of Food Science and Nutritional Engineering, China Agricultural University, Beijing, 100083, China; College of Food Science and Nutritional Engineering, China Agricultural University, Beijing, 100083, China; Institute for Soil, Water and Environmental Sciences, Agricultural Research Organization, Ramat Yishay 30095, Israel; Department of Water Resources and Architectural Engineering, Beijing Vocational College of Agriculture, Beijing 102208, China; State Key Laboratory of Efficient Utilization of Agricultural Water Resources, China Agricultural University, Beijing 100083, China; Engineering Research Center for Agricultural Water-Saving and Water Resources, Ministry of Education, China Agricultural University, Beijing 100083, China

## Abstract

Eating fruits and vegetables loaded with natural antioxidants can boost human health considerably and help fight off diseases linked to oxidative stress. Hydrogen has unique antioxidant effects. However, its low-solubility and fast-diffusion has limited its applications in agriculture. Integration of hydrogen with nanobubble technology could address such problems. However, the physiological adaptation and response mechanism of crops to hydrogen nanobubbles is still poorly understood. Antioxidant concentrations of lycopene, ascorbic acid, flavonoids, and resveratrol in hydrogen nanobubble water drip-irrigated tomato fruits increased by 16.3–264.8% and 2.2–19.8%, respectively, compared to underground water and oxygen nanobubble water. Transcriptomic and metabolomic analyses were combined to investigate the regulatory mechanisms that differed from the controls. Comprehensive multi-omics analysis revealed differences in the abundances of genes responsible for hormonal control, hydrogenase genes, and necessary synthetic metabolites of antioxidants, which helped to clarify the observed improvements in antioxidants. This is the first case of hydrogen nanobubble water irrigation increasing numerous natural antioxidant parts in fruits. Considering the characteristics of hydrogen and the application of the nanobubble technology in agriculture, the findings of the present study could facilitate the understanding of the potential effects of hydrogen on biological processes and the mechanisms of action on plant growth and development.

## Introduction

Under aerobic conditions, plant activities are influenced considerably by reactive oxygen species (ROS) metabolism. At low concentrations, ROS may act as signaling molecules triggering gene expression to regulate plant resilience [1]. ROS activates the mitogen-activated protein kinases (MAPKs) cascade in response to stress, which is coordinated with signaling changes that trigger stress-specific signaling pathways, such as redox signaling. Subsequently, the signaling molecule can be transported through the electron transport chain in mitochondria to the nucleus to trigger gene expression. The activation of signaling pathways is also accompanied by a rapid increase in hormone levels, such as newly synthesized jasmonic acid, or the release of abscisic and salicylic acids. However, excessive ROS leads to oxidative stress, and damages cells, tissues, and organs. Therefore, a rich tapestry of antioxidant molecules in plants plays a vital role in maintaining a balance between removal or regulation of ROS.

The non-enzymatic antioxidants of plants, such as vitamins, glutathione, carotenoids, flavonoids, and resveratrol help reduce oxidative stress-related damage by efficiently utilizing different high-capacity reductases that enable them to effectively regulate the cellular redox status [2, 3], which guarantees the nutritional value of plant foods. Research has demonstrated that the antioxidant activity of phytonutrients, such as ascorbic acid and carotenoids is reduced by nearly 50% after being subjected to intestinal digestion, with a significant decrease in the average concentration of bioactive compounds [4]. Furthermore, a minimum daily intake of 400–500 g of fruits and vegetables has been recommended by the World Health Organization for the prevention of hypertension, stroke, and cardiovascular disease and amelioration of renal disease and other micronutrient-related deficiencies [5–7].

Several studies have identified effective methods of increasing the concentrations of natural antioxidant components in fruits and vegetables. For example, applying microbial inoculum to the soil, applying micronutrient fertilizers, and controlling soil moisture in the root zone have been demonstrated to increase the concentrations of specific antioxidants, such as lycopene (LYC) or ascorbic acid (AsA) [8, 9]. However, the use of high concentrations of microbial inoculum, large amounts of trace elements, and excessive water control could increase the risk of decline in soil health, crop wilting, and reduced yields [10, 11].

Recent studies have demonstrated that hydrogen, owing to its reduction activity, can be used to increase plant tolerance to salt, drought, and heavy metal stress via the promotion of the activity of antioxidant enzymes, such as ascorbate peroxidase (APX) and CATalase (CAT) and regulation of the expression of genes related to glutathione metabolism [12–14]. Plants treated with hydrogen-rich water have reportedly higher antioxidant activity, lower lipid peroxidation levels, prolonged radical scavenging activity, and increased ·OH and ·DPPH scavenging activity [15]. In addition, exogenous pretreatments with hydrogen-rich water differentially attenuate the inhibition of seed germination and seedling growth caused by salinity [16]. Furthermore, hydrogen-rich water positively affects the abundance of flavor components such as esters (e.g., furanone derivative relative concentrations increased 10.8- and 13.2-fold in strawberries irrigated with experimental treatments, respectively) and soluble sugars (e.g., glucose, fructose, and sucrose) in fruits, including strawberries, as well as in postharvest horticultural products [12, 15]. However, the applicability of hydrogen-rich water in improving agricultural productivity is hindered by the low solubility and strong diffusivity of hydrogen [17].

Nanobubbles (<1 μm in diameter) have become a hot research topic because of the inherent characteristics that can efficiently enhance the solubility and retention duration of gases in liquids owing to their excellent mass transfer, high surface tension, and ability to implode and generate free radicals [18]. In addition, nanobubble irrigation technology has been shown to increase agricultural productivity and improve crop quality. Some researchers have observed that oxygen nanobubble (ONBs) water irrigation enhances O_2_ delivery to soil, improves organic fertilizer utilization by soil microbes, promotes aerobic respiration and ROS concentration by plants, activates plant proliferative pathways, such as flavor quality; for example, vitamins (e.g., AsA) and soluble sugars were improved significantly in tomatoes, watermelons, and cucumbers [19, 20]. However, ONBs could promote ROS by generating hydroxyl radicals through the collapse of nanobubbles. As the gas inside nanobubbles escapes and is consumed, stimulation of redox reactions in the model organism cannot be sustained [21]. Therefore, low concentrations of nanobubbles are considered to promote the metabolic ability and antioxidant capacity of plants by increasing antioxidant enzyme activity [22]. Although the properties of nanobubbles make the technology the ideal choice for use in the preparation of hydrogen-rich water, the impact of hydrogen nanobubbles (HNBs) irrigation technology on crop antioxidants and the mechanisms underlying its effects have not yet been sufficiently elucidated.

Here, we explore the potential of subsurface drip irrigation with water enriched with HNBs to improve the levels of antioxidant compounds in tomato fruits. Based on the ability of hydrogen gas to selectively scavenge free radicals, we hypothesized that HNBs irrigation would modulate the electron transport chain signaling and promote transcript abundance in the non-enzymatic antioxidant biosynthesis pathway, thereby increasing antioxidant concentrations in tomato fruits.

## Results

### Effect of drip irrigation with HNBs on antioxidants and flavor quality of tomato fruits

**Figure 1:**
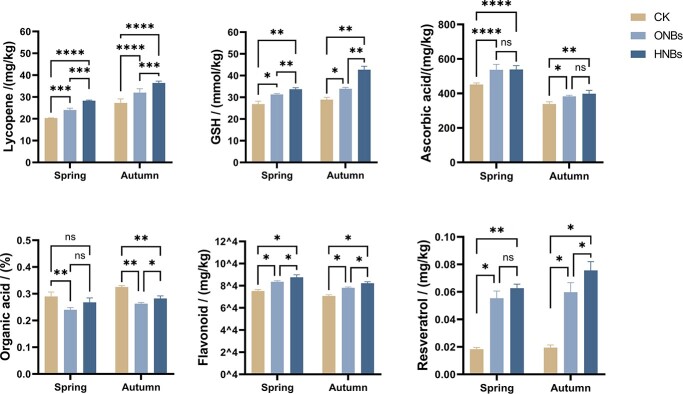
Antioxidant concentrations in tomato under different nanobubble irrigation treatments. The bar graphs illustrate differences in lycopene, glutathione, ascorbic acid, organic acids, flavonoids, and resveratrol among the three groups of fruits in spring and autumn of the same year. *n* = 6 replicates; *P*-value style is GP: *P* = 0.1234 [not significant (ns)], ^*^P = 0.0332, ^**^P = 0.0021, ^***^P = 0.0002, and ^****^P < 0.0001. Data are mean ± standard error (SE).

Throughout the two-season growing periods, the plants in the greenhouse were irrigated with groundwater/nanobubble water from the seedling stage. Compared with groundwater drip irrigation (CK), irrigation with nanobubble water from both gas sources (ONBs and HNBs) enhanced the antioxidant concentrations in the fruits and decreased organic acid concentrations significantly (*P <* 0.01; Fig. 1). HNBs increased the average LYC and glutathione (GSH) concentration in the fruits significantly compared to the CK and ONBs (LYC: 36.1% and 15.9%, respectively; GSH: 36.8% and 16.8%, respectively). The polyphenol resveratrol was also improved significantly by nanobubble irrigation, and the increase was greater by 19.8% for HNBs than for ONBs. The average AsA and flavonoid concentrations in HNB-treated tomato fruits were elevated significantly, by 18.5% and 16.3%, respectively, compared to those in CK. The organic acid concentration of tomato fruits in the fall decreased, with a greater reduction in the ONBs compared to in the HNBs (9.5% higher).

**Figure 2:**
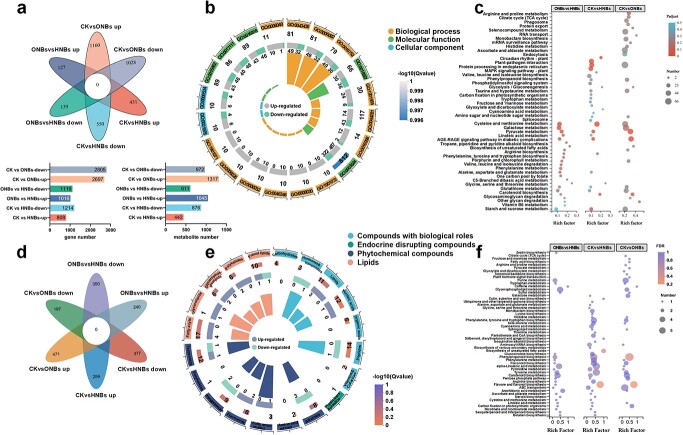
Effects of treatment groups (CK, ONBs, HNBs) on the transcriptome and metabolome of tomato fruit. **a** Venn diagram showing the different genes. **b** Gene Ontology (GO) functional enrichment circle graph shows the differences in gene expression (ONBs and HNBs). **c** KEGG pathway enrichment bubble group graphs of the differentially expressed genes. **d** Metabolite Venn diagram. **e** KEGG functional enrichment circle graph showing the difference in metabolites between the two experimental groups (ONBs vs HNBs). **f** Bubble group graph representing the KEGG pathway enrichment of distinct metabolites.

**Figure 3:**
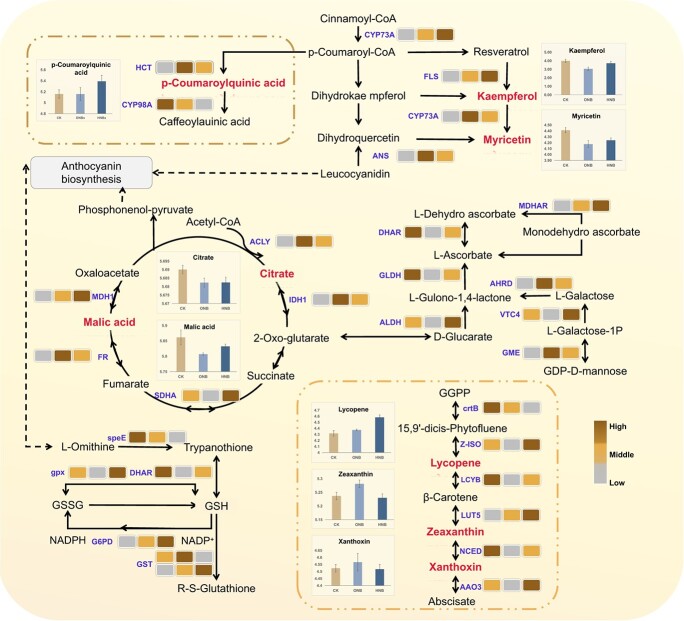
Impact of HNBs and ONBs on the interactive signal transduction process of antioxidant quality in fruits. In sequence, the boxes represent gene expression under the CK, ONB, and HNB treatments; the color of the legend indicates the relative expression level of genes between groups after standardization treatment in the sample; names in purple fonts represent enzymes; the red bold font indicates the differential metabolites discovered during the experiment; the bars represent the expression abundances of the differential metabolites.

**Figure 4:**
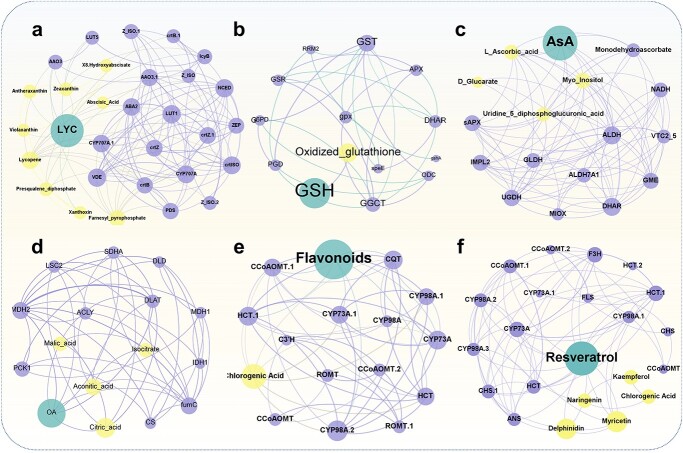
Network relationship between antioxidant quality, gene expression and metabolites upon HNB drip irrigation. The nodes in the network plot indicate different genes, metabolites, and fruit phenotypic data. Nodes of the same color indicate the same categorization (purple for genes, yellow for metabolites, and green for phenotypes). Connection reflects whether there is a positive or negative association between this data and other data based on its connectedness (purple shows positive correlations, green shows negative correlations, and thickness indicates the size of the weights).

### Gene expression and metabolite in HNBs fruit show dramatic variation in general

To analyse the effect of the nanobubbles in the irrigation water on the antioxidant quality of fruits, transcriptome and metabolomic analyses of the tomato fruits were performed (Fig. 2). Principal component analysis using the transcriptome data revealed that the nanobubble irrigation had differential effects on the transcriptomic profile of the tomato fruits (Fig. S1a, see online supplementary material). According to the pairwise comparison, compared with the CK, HNBs upregulated 808 genes (Fig. 2a). In total, 2132 differentially expressed genes (DEGs) were identified between the ONBs and HNBs, among which 1016 showed significantly enhanced responses to HNB treatment. Functional classification of the DEGs with 1.5-fold change (FC) by mapping the annotation data obtained from the Gene Ontology (GO) database revealed that most DEGs are involved in biological regulation and cellular processes of biological process and involved in the cell part and organelle of molecular function. The DEGs also have transporter activity and catalytic activity (Fig. 2b; Fig. S1b and c, see online supplementary material). Differential gene enrichment in membrane component function was distinct across the upregulated and downregulated genes, which may represent a variation in the response caused by the experimental treatment. According to the results of Kyoto Encyclopedia of Genes and Genomes (KEGG) pathway enrichment analyses of the three groups of DEGs, the differential response changes in various pathways induced by the experimental treatments were enriched significantly in fatty acid degradation, GSH metabolism, phenylpropanoid biosynthesis, terpenoid biosynthesis, and MAPK signal transduction pathways (Fig. 2c). Additionally, terpenoids generally have significant antioxidant activity, and the biosynthetic pathways of GSH and phenylpropanoids can directly produce antioxidants or their precursors, whereas lipid peroxidation produced during lipid metabolism damages cells and can lead to accelerated aging. The results indicated that nanobubbles and gas in nanobubbles play crucial roles in signaling during fruit development.

Based on the differences in gene expression in fresh tissue, the metabolites in the same samples were analysed. Consistent with the transcriptomic analysis results, the metabolomes of different experimental treatments showed considerable differences (Fig. S1d, see online supplementary material). Venn diagram analysis of the differential substances derived from the pairwise comparative analyses of the three treatment groups showed that specific responses occurred for 668 and 636 substances in ONB and HNB, respectively, compared with CK. Additionally, specific responses were measured for 590 substances when comparing ONBs and HNBs (Fig. 2d). KEGG compound categorization statistics (Fig. 2e) were calculated by comparing the KEGG database for all discovered metabolites (VIP >1, *P* < 0.05). The KEGG pathway enrichment analysis of all differential metabolites (Fig. 2f; Fig. S1e and f, see online supplementary material) revealed 54 pathways, most of which were involved in tyrosine metabolism, phenylpropanoid biosynthesis, histidine metabolism, flavonoid and flavonol biosynthesis, and carotenoid biosynthesis. The synthesis pathway of flavonoid and carotenoid determine the concentration of quality directly. In addition, tyrosine and lysine have strong antioxidant capacity, and their intermediate metabolites can participate in the promotion of the oxidative metabolism process in cells or the catalytic reactions of various redox enzymes. The results were comparable to those of differential gene enrichment in the KEGG pathway and demonstrated that HNBs had a stronger influence than ONBs on the biosynthesis of most chemicals in the fruits.

### HNBs drip irrigation influenced gene expression and metabolite abundance in tomato functional quality

To explore the response mechanism of HNB drip irrigation treatment on the functional quality of tomato fruits, we integrated multi-omics data of the samples. Using the KEGG pathway analysis, the pathways of metabolites and genes associated with each quality indicator that exhibited a FC were mapped (Fig. 3). The results from quantitative real-time reverse transcription PCR (qRT-PCR) (Fig. S2a, see online supplementary material) experiments demonstrated that nanobubble treatment altered the expression of the genes linked to the AsA-reduced GSH conversion processes significantly. HNBs had a considerably higher abundance of monodehydroascorbate reductase (MDHAR) and glutathione S-transferase (GST) than ONBs. Nanobubble water irrigation positively affected the abundances of enzymes such as GDP-mannose-3′,5′-epimerase (GME), and aldehyde dehydrogenase (AHRD) in the AsA synthesis pathway and the key enzyme *MDHAR* of the regenerative cycle. Along with the aforementioned genes, treatment with HNBs upregulated the *MDHAR* and *GME* pathway more significantly than that with ONBs. Furthermore, metabolomic analysis revealed that the LYC concentration of HNBs was significantly improved by 15.9% more than that of ONBs, owing to HNBs increasing significantly the expression of violaxanthin de-epoxidase (*VDE*) related to abscisic acid synthesis in the carotenoid biosynthesis pathway. In addition, the abundance of intermediate differential metabolites in abscisic acid synthesis revealed a trend of higher oxygen abundance than hydrogen abundance. The qRT-PCR and metabolome data confirmed that the LYC concentrations of HNBs and ONBs tomatoes were significantly different (Fig. S2a, see online supplementary material), which was explained by the significant upregulation of LYC production-related genes.

Compared to ONBs, the expression levels of genes associated with the enzymatic production of caffeoylquinic acid, an upstream component of HNBs, increased dramatically. After exposure to nanobubbles, the major enzymes involved in flavonoid metabolism and resveratrol, trans-cinnamate 4-monooxygenase (*CYP73A*), and flavonol synthase (*FLS*) pathways in the fruits were downregulated significantly. The tricarboxylic acid (TCA) cycle is the main source of energy production in plants. In the present study, treatment with nanobubble water drastically reduced the amount of malate accumulated in the cycle. Furthermore, our transcriptomic data revealed that the CK and HNBs dramatically increased the expression of enzymes involved in organic acid production.

### Relationships between antioxidants, genes, and metabolites of tomato in HNBs

Integration of whole antioxidant data, including concentration, gene expression, and metabolite abundance in correlation analysis could identify elements for antioxidant quality improvement. Subsequently, Pearson correlation analysis was used and a network of phenotypic information, transcripts, and metabolites was constructed for each antioxidant. A total of 110 differential genes (FC >1.5) enriched in six biosynthetic pathways and phenotypic data of six antioxidant molecules in three groups of experimental fruit samples were analysed visually (Fig. 4, Spearman, *P*_adjust_ < 0.05, Benjamini–Hochberg method). The results showed substantial correlation among the genes, metabolites, and phenotypes in the fruits of the plants irrigated with HNBs. The qRT-PCR analysis (Fig. S2b, see online supplementary material) demonstrated that the expression of aldehyde oxidase 3 (*AAO3*), β-ring hydroxylase (*LUT5*) and β-carotene hydroxylase-2 (*BCH2*) genes, as well as the amount of zeaxanthin and LYC, were associated with LYC accumulation (Fig. 4). In contrast, the accumulation of oxidized GSH was closely related to GSH synthesis. The increase in AsA concentration was positively correlated with the expression of six genes [*ALDH, MDHAR, GME, VTC 4, DHAR,* glutamic dehydrogenase (*GLDH*)] and three metabolites (L-ascorbic acid, D-glucarate, and myoinositol). The formation of organic acid was correlated with citric acid concentration and expression of six genes [hydratase class II (*fumC*), malate dehydrogenase (*MDH2*), dihydrolipoyl dehydrogenase (*DLD*), citrate synthase (*CS*), isocitrate dehydrogenase (*IDH1*), and phosphoenolpyruvate carboxy kinase 1 (*PCK1*)]*.* In contrast*,* the expression *CYP73A,* hydroxycinnamic acid transferase (*HCT*), carnitine octanoyltransferase (*CQT*), coumarin-3-hydroxylase (*C3H*), 5-O-(4-coumaroyl)-D-quinate 3′-monooxygenase (*CYP98A*), trans-resveratrol di-O-methyltransferase (*ROMT*), and caffeoyl-CoA O-methyltransferase (*CCoAOMT*) was correlated significantly with the development of the flavonoid synthesis pathway. However, the production of resveratrol in fruits was associated with the presence of delphinidin and myricetin, as well as the expression *F3H*, Chalcone synthase (*CHS*), anthocyanidin synthase (*ANS*), *FLS*, *CYP73A*, *HCT*, *CYP98A*, and *CCoAOMT*.

## Discussion

### The differential mechanism by which HNBs and ONBs modulate tomato antioxidant quality

Fruits obtained from HNBs, as opposed to ONBs, had much higher levels of antioxidant components, such as LYC, GSH, and resveratrol. Combined with the KEGG pathway enrichment analysis of DEGs between HNBs and ONBs, we discovered that unlike HNBs, ONBs controlled fruit metabolic processes by modulating phytohormone signaling mechanisms and significantly activating genes associated with growth hormone-inducible proteins (Fig. S3, see online supplementary material). Hydrogenases also aid plants in the conversion and uptake of intracellular hydrogen [23]. qRT-PCR revealed that HNBs significantly affected the genes encoding the enzymes involved in oxidative phosphorylation, lysine, and other amino acid biosynthetic pathways (Fig. 2c; Fig. S4, see online supplementary material). The electron transport chain utilizes a series of electron transfer reactions to generate cellular ATP through oxidative phosphorylation accompanying the generation of ROS [24]. HNBs significantly increased the expression of ATP-producing genes, such as *ATPeV_1_E*, *ATPeF_1_B*, and *ATPeF_0_D* (Fig. S4a, see online supplementary material) while suppressing the genes for the chromoproteins *Cytb* and *COX1* using iron porphyrin as a cofactor (Fig. S4b, see online supplementary material). This may be due to reversible changes in the valence state of the iron atoms in the heme cofactor of the cytochrome enzyme complex, which allows the complex to function as an electron carrier in the respiratory chain. In HNBs, hydrogen gas or hydrogen atoms carried into the plant body as nanobubbles could have altered the activity of the enzymes, resulting in downregulated expression of related genes [25]. However, by modifying the microenvironment around tryptophan (Trp), hydrogen bonding was enhanced, reducing the distance between Trp and the heme center and increasing the efficiency of energy transfer from cytochrome oxidase to the active center with enzyme activity [1, 26, 27]. SDHA is a component of the enzyme succinate dehydrogenase complex (SDH) in the TCA cycle. The results of the transcriptomic data suggest that *SDHA* was up-regulated, with significant up-regulation of *MDH1* downstream, which might accelerate the dehydrogenation of succinate to form fumarate and decrease the consumption of fumarate, leading to fumarate accumulation. Fumarate can continue to perform mitochondrial electron transport functions as a terminal electron acceptor [28]. NADH dehydrogenase-related genes, such as *NDUFS1* and *NDUFA8 (*Fig. S5, see online supplementary material)*,* were upregulated in HNB fruits significantly compared with ONBs, suggesting that HNBs stimulate and positively regulate NADH synthesis in cells, resulting in NADH regeneration, which improves the efficiency of electron transfer in fruits while promoting energy accumulation [29, 30]. In general, the results indicated that respiratory activity and structural organization of functional mitochondrial respiratory supercomplexes vary with HNBs and might effect F-type H^+^-transporting ATPase expression directly to improve ATP content without producing extra ROS.

### Mechanism of the synergistic enhancement of the multiple antioxidant properties of tomato by HNBs

Understanding how HNBs affect the antioxidant quality of fruits is critical for understanding the interactions between hydrogen and nanobubbles. Correlation analysis of the DEGs enriched in the biosynthetic pathways of antioxidants, such as carotenoids, AsA, GSH, flavonoids, resveratrol, and organic acids, revealed physical correlation among DEGs, even among different pathways (Fig. S6, see online supplementary material, r > 0.75). The network of DEGs with metabolite and phenotype data suggests that by gradually changing the expression of differential metabolites, several significantly altered differential genes may eventually increase the total amount of each antioxidant molecule. HNBs upregulated *LCYB* and *NCED*, which confer nutrient accumulation and improve crop tolerance to abiotic stresses by regulating the distribution ratio of carotene and lutein in fruit, which might explain the carotenoid synthesis promotion effect [31, 32]. In addition, HNBs with a high linkage number upregulated the expression of *AAO3* and *CYP707A1*, the catabolic enzymes of ABA, significantly. The data indicate that HNBs might modulate carotenoid composition and concentration by controlling ABA metabolism while preventing excessive accumulation [33, 34]. HNBs had a significant impact on the reduced and oxidized GSH cycle pathways because hydrogen functions as a signaling molecule that influences nitric oxide-related enzyme activity to regulate sulfhydryl groups in protein synthesis [23] within cysteine and methionine, thereby increasing the rate of amino acid synthesis and providing sufficient substrates for GSH synthesis (Fig. 4).

In the ascorbate regeneration pathway, AsA and dehydroascorbic acid were interconverted under the control of *DHAR*, which was upregulated dramatically during HNBs. This implies that the plant improved the AsA regeneration cycle while decreasing the negative effects of oxidative stress on the environment, thereby increasing the AsA concentration [35, 36]. ACLY, IDH1, LSC2, and MDH1 are key enzymes involved in the synthesis of ferredoxin and oxaloacetate [37] MDH1 is a critical enzyme involved in malate production. However, the NAD-meME upregulated expression and downregulated in malate implied that HNBs might have expedited malate oxidation by enhancing its enzymatic activity, resulting in a decrease in acidity. HNBs dramatically upregulated *MDH1*, which might enhance the abundance of fructose and sucrose. Significantly upregulated expression of *CYP73A* in the flavonoid-generating pathway after HNBs effectively decreased lignification and increased flavonoid production [38, 39]. Furthermore, the enzyme-encoding genes in this pathway, including *CYP98A* and *CCoAOMT*, have multiple relationships with other genes, metabolites, and symptoms. The genes have equivalent linkage strengths in the biosynthetic pathway that generate resveratrol. This demonstrates that the response of a single gene to HNBs might play a role in several biosynthetic pathways to achieve convergent regulation of multiple antioxidant properties, which is consistent with the findings of a previous study in rice [40].

**Figure 5:**
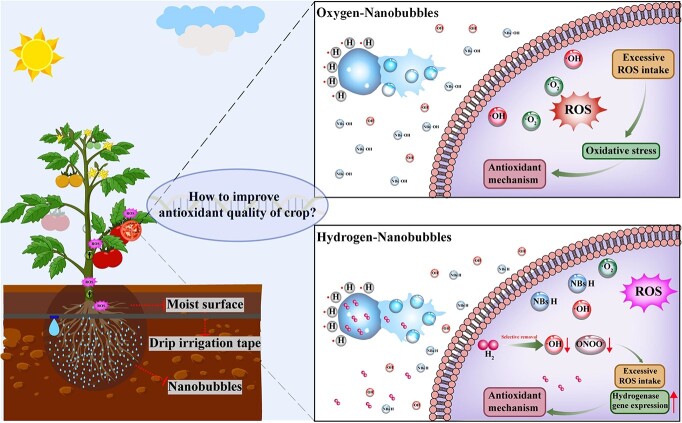
Conceptual figure explaining the impact of HNBs on the antioxidant quality of tomatoes. (i) ONBs may use the free radicals produced by bubble rupture to induce the antioxidant mechanism, and (ii) HNBs may promote the expression of genes, such as those encoding for hydrogenase, to increase the accumulation of natural antioxidants.

In summary, we concluded that the mechanism of action of HNBs of increasing the antioxidant potential of fruits is summarized in Fig. 5. The trace exogenous ROS created by HNBs transported into the plant structure impacted the antioxidant defense mechanism in plants without utilizing too many native antioxidant components. Moreover, the hydrogen molecules in the nanobubbles may minimize the negative effects of ROS and co-promote the accumulation of antioxidant molecules in the fruit by stimulating the efficiency of electron transfer as another antioxidant defense pathway.

### Antioxidant quality regulation mechanism of tomato nanobubble water irrigation

Subsurface drip irrigation (SDI) with groundwater enriched with nanobubbles (nanobubble water) increased the concentrations of GSH, AsA, carotenoids, flavonoids, and resveratrol by at least approximately 10% in fruits compared to in the CK. The phenotypic difference led us to analyse the relationship between the transcriptomic and metabolitein of fruit. Our findings revealed that nanobubble water irrigation significantly increased the expression of numerous essential genes associated with the six antioxidant properties, which affected the abundance of adjacent metabolites. It has been reported that alterations in a single component do not control all aspects of plant life [41]. The ROS generated by their rupture may affect hormone production by interfering with photosynthetic capacity or acting as signaling molecules to activate their antioxidant systems to combat ROS-induced oxidative stress and maintain the redox balance *in vivo* [2, 42]. Consequently, numerous hormones may be involved in responses to the mobility of small nanobubbles under intracellular pressure in plants. The ·OH content, as the main component of ROS generation after nanobubble rupture, compared to CK, increased by 9.0% with HNBs and 35.8% with ONBs (Fig. S7, see online supplementary material). This indicated that a certain amount of nanobubbles are present in the fruit. Enzymes that produce and scavenge ROS were key to regulating ROS as second messengers, and the resulting changes in ROS can lead to the oxidation of transcription factors and enzymes that alter hormonal and developmental pathways for plant growth and developmental signaling [29]. Jasmonic acid is known to control ROS concentration through increased oxidase levels and activity [42, 43]. In addition, the ROS might mediate biosynthesis and gene expression of ABA directly to activate resistance [32]. However, expression of ABA2 as one of the seven genes involved in ABA synthesis could influence its concentration [27]. In addition, BIN2 regulates jasmonic acid content and influences the expression of responsive marker genes [40]. The qRT-PCR results confirmed that in the nanobubble water irrigation conditions, oleuropein lactone *BIN2*, and *ABA2,* which are the key enzyme genes involved in the signaling pathways of jasmonic acid and abscisic acid, were upregulated significantly by 4.0–6.3, and 9.6–12.0-fold, respectively, when compared with the CK (Fig. S8a, see online supplementary material). Accordingly, ABA and jasmonic acid concentrations were increased 1.2–1.3 and 1.1–1.4 fold, respectively (Fig. S8b, see online supplementary material). The results suggested that several hormones may respond to ROS generated by the bursting of nanobubbles in plants, regulating resistance-related activities. Furthermore, combined with other hormones, it could synergistically enhance the function of plant antioxidant systems [5, 44]. Antioxidant concentrations of lycopene, ascorbic acid, flavonoids, and resveratrol in hydrogen nanobubble water drip-irrigated tomato fruits increased by 16.3–264.8% and 2.2–19.8%, respectively, compared to underground water and oxygen nanobubble water [45].

Additionally, we found that in the ascorbate and aldarate metabolism, differential genes such as dehydroascorbate reductase and *GLDH* showed the same down-regulated trend in HNBs and ONBs compared to CK and, during the degradation of resveratrol, there was no significant difference between HNBs and ONBs in gene abundance and metabolite Myricetin such as *FLS* and *CYP73A*. This may be the reason for the non-significant difference in concentration between ascorbic acid and resveratrol between the ONBs and HNBs.

### Prospects for HNB technology application.

Irrigation with nanobubble water is a novel type of aeration technology for the irrigation water source that could synergistically improve crop yield and quality [13, 46]. In the present study, HNBs demonstrated a similar conclusion; it simultaneously enhanced the concentrations of several antioxidants, the antioxidant capacity (Fig. S9, see online supplementary material) of tomato and yield were increased significantly (Fig. S10, see online supplementary material). Enzyme activities, such as superoxide dismutase (SOD), glutathione peroxidase (GSH-Px), and ascorbate peroxidase (APX) of HNBs were 37.6%, 50.1%, and 64.5% higher than those of CK, respectively. The scavenging capacities of hydroxyl (·OH), 2,2′-azino-bis (3-ethylbenzothiazoline-6-sulfonic acid) (ABTS), and 2,2-diphenyl-1- picrylhydrazyl (·DPPH) radicals were significantly greater than that of CK by 65.5%, 32.8% and 29.7%, respectively. The yield increased by 22.5% on average compared with CK. As a result, in-depth research into HNBs can hasten the adoption of hydrogen agriculture and the extension of irrigation technologies, such as determining the optimal hydrogen requirements of plants at different developmental stages.

However, the optimal hydrogen requirements of different crops at different developmental stages, the optimal frequency of HNB irrigation, and the optimal concentration of hydrogen applied are yet to be studied comprehensively, which restrict the sustainable development and promotion of HNB technology for the precise regulation and control of the yields of agricultural commodities to some extent. Therefore, such subjects should be the focus of future research to improve and accelerate the application of HNB irrigation technology in agriculture.

## Conclusions

This is the first study to report that HNB subsurface drip irrigation of tomatoes significantly affects fruit natural antioxidant qualities. Multi-omics analysis with transcriptomics and metabolomics was used to explore the response mechanisms of fruit to HNB irrigation. Based on the differences in gene abundance of hydrogenase-related genes on the electron transport chain in the multi-omics analysis, we inferred that HNBs particularly regulated it. Meanwhile, hormone biosynthesis-related gene abundance, antioxidant biosynthesis-related gene abundance, and differences in metabolite concentrations among groups clarified the reasons for the observed significant elevation of antioxidants. Our study verifies a new technology for enhancing phenotypic plasticity in horticultural crops and demonstrates the potential of HNB technology to promote biological processes (e.g., as yield and food quality). The research enriches our understanding of the mechanism of action of HNB, which could facilitate plant growth and development.

## Materials and methods

### Experimental design

Spring (March – June) and fall (July – October) tomato (*Solanum lycopersicum*) growth experiments were carried out at the greenhouse of the Tongzhou Experimental Station of China.

All treatments were irrigated using SDI systems. The treatments were arranged as follows: CK as the control group; groundwater enriched by ONBs as the positive control, and groundwater enriched by HNBs as the second experimental group. Each treatment had four replicates with plots measuring 1.5 × 6 m^2^, containing 30 tomato plants and two drip laterals. Drip tape emitters were spaced 40 cm apart and buried at 15 cm, with a flow rate of 2.6 L/h.

During the entire growth period, plants were irrigated 18 times. The total irrigation volume was 3915 m^3^/ha. Comprehensive descriptions of growing conditions and irrigation/fertilization regimes for the tomato plants are presented in Methods S1 (Table S1, see online supplementary material).

A nanobubble generator, developed through independent research and development, with an intake flow rate of 3 L/min, was connected to the SDI system. It had an average bubble size and density of 136.2 ± 12.1 nm and 6.2 ± 10^8^ bubbles/mL, respectively, determined using a NanoSight 300 analyzer (Malvern, UK). The volume and purity (>99.99%) of hydrogen and oxygen used to produce the nanobubbles were the same. The hydrogen gas was bubbled into 10 L underground water at a rate of 3 Nl/min for 20 min.

### Sample collection

Tomato fruit samples collected on the third day of the ripening stage had three and six biological replicates of flesh tissues immediately frozen in liquid nitrogen for RNA isolation and LC–MS measurements, respectively, and stored at −80°C. Each biological replicate was derived from the same sampling regions (one-quarter of the middle) from three tomatoes [35].

### AsA analysis

Each sample (10 g) was crushed and extracted three times with 5% (w/v) trichloroacetic acid. The mixtures were obtained by centrifugation and incubation, and concentrations (y) were determined spectrophotometrically at 534 nm [20] using a calibration curve with AsA standards (y = 0.0963x − 0.0444; R^2^ = 0.9989). The detailed preparation process is presented in Methods S2 (Table S2, see online supplementary material).

### Organic acid analysis

Ten grams of each material were ground in a mortar and the mixture was incubated at 25–28°C for 30 min. The filtrate (20 mL) was aspirated into a triangular flask with two drops of 1% phenolphthalein indicator and titrated with 0.1 mol/L sodium hydroxide solution. The amount of titrated sodium hydroxide was recorded. Because the malate was the predominant acid of tomato, 0.067 was chosen as the conversion factor [47].

### LYC analysis

LYC was extracted from one gram of fresh tissue and then filtered through a 0.22-μm membrane (Sigma-Aldrich, St Louis, MO, USA), and the compounds were separated using an HPLC1200 system with a C18 reverse phase column (0.5 μm, 250 × 4.6 mm). The LYC peak was analysed at 445 nm, and a calibration curve was constructed using LYC standards (y = 28.204x − 11.443; R^2^ = 0.9996) for identification and quantification [1]. The preparation process is presented in detail in Methods S3 (see online supplementary material).

### Flavonoids analysis

Two grams of tissue were ground with 1% HCl-methanol solution in a pre-cooled mortar three times. The solutions were transferred to a 20-mL graduated test tube and left in a light-proof environment at 4°C for 20 min. The flavonoid concentration of the filtrate was determined spectrophotometrically at 325 nm [47] and calculated using a calibration curve with Rutin standards (y = 0.0122x + 0.0018; R^2^ = 0.9981). The preparation process for the calibration curve is shown in Methods S4 (Table S3, see online supplementary material).

### Glutathione (GSH) analysis

Glutathione (GSH) was extracted through by grinding and centrifuging 5 g of the sample. The GSH concentration was measured at 412 nm and calculated using a calibration curve with GSH standards (y = 0.1209x; R^2^ = 0.9821) [45]. The preparation process is presented in detail in Methods S5 (Table S4, see online supplementary material).

### Resveratrol analysis

Resveratrol was extracted by centrifugation and sonicated from 5 g of a sample. The supernatants were pooled and filtered using a 0.22-μm filter membrane for further use. Compounds were separated using a HPLC1200 system fitted with a C18 reverse phase HPLC column (0.5 μm, 250 × 4.6 mm). The resveratrol peak was analysed at 290 nm, and the calibration curve was developed using resveratrol standards (y = 87.237x − 1.6619; R^2^ = 0.9996) for identification [30]. The preparation process is presented in detail in Methods S6 (see online supplementary material).

### Transcriptome sequencing and data analysis

Total RNA was extracted from fresh tomato tissue (see Sample Collection section) using TRIzol (Invitrogen). Libraries were sequenced using high-throughput Illumina NovaSeq 6000 sequencing technology with a paired-end read length of 2 × 150 bp [48]. The raw paired-end reads were trimmed and quality controlled by SeqPrep and Sickle with default parameters. The sequencing depth was >6G, and the Q30 contents was >94.1%. The raw data were mapped to the tomato reference genome Solanum_lycopersicum4.0 (https://www.solgenomics.net/organism/Solanum_lycopersicum/genome/) using HiSat2. The mapped reads of each sample were assembled by StringTie in a reference-based approach. DEGs were screened using DESeq2 with filter criteria of fold change (FC) ≥1.5 and adjusted *P*-value (*P*-adjust) of <0.05.

### Validation of selected DEGs by quantitative real-time reverse transcription PCR

The expression of eight representative genes from antioxidant-biosynthetic pathways was assessed in the tomato flesh tissues. qRT-PCR was performed using SYBR Green PCR Master Mix with a real-time PCR System CFX96 (Bio-Rad, CA, USA). Relative expression was determined using the primers and steps listed in Methods S7 (Table S5, see online supplementary material) [49].

### Metabolite identification using LC–MS and data analysis

Frozen solid samples (50 mg) were extracted using methanol: water (4:1; v:v) and 0.02 mg/mL L-2 chlorophenylalanine, crushed (−10°C, 50 Hz), centrifuged (13 000 *g*, 15 min, 4°C), and transferred to a chromatographic column for mass spectrometric detection (UHPLC-Q Exactive HF-X, Thermo Fisher Scientific). Significant differences in metabolite levels between the control and the two treatments were identified using the criteria of *P*-value (<0.05) and variable importance in projection (VIP; >1) relative to the control. Supervised partial least squares discriminant analysis (PLS-DA) was performed to obtain a global overview of metabolic changes [5].

### Data analysis and visualization

Statistical analyses were both performed in GraphPad Prism 9 (GraphPad Software Inc., Boston, MA, USA) by one-way or two-way analysis of variance (ANOVA) followed by Bonferroni multiple comparisons test, and *P*-value style is GP: *P* = 0.1234 [not significant (ns)], ^*^P = 0.0332, ^**^P = 0.0021, ^***^P = 0.0002, and ^****^P < 0.0001.

## Acknowledgements

This study was financially supported by the Major Program of National Natural Science Foundation of China (52339004) and National Natural Science Foundation of China (51979274 and 52109070). We thank the Deutsche Forschungsgemeinschaft (DFG, German Research Foundation) for funding: Projektnummer 471624304. We thank Dr David Midmore for language editing, Dr Sergi Munné-Bosch for recommending and guiding the advanced experimental methods, Abidur Rahman for suggesting transcriptome testing and hormone results validation. We thank Majorbio for providing the platform for gene sequencing, metabolite identification, and Majorbio Cloud Platform. We thank Shanghai Yuanye Bio-Technology Co., Ltd and Beijing Solarbio Science & Technology Co., Ltd for providing test kits for enzyme activity and radical scavenging capacity to assay antioxidant capacity of tomatoes.

## Author contributions

Y.L. conceived and designed the research. J.H. conducted the experiments, analysed the data, and wrote the paper. Y.L. and Y.Z. gave guidance on the paper structure and picture design. Y.Z. and C.-M.G. checked and enhanced the grammar and descriptions, and improved paragraph coherence and enriched data. J.C. guided on the methodology during the experiments, and then checked and modified the description of the Materials and Methods of the paper. D.F. provided a prominent contribution on the pictures’ details and multiomics professional expression. S.B. meticulously checked and revised the descriptions of the results and graphical details. Y.L. and Y.L. discussed the results and commented on the manuscript.

## Data availability

The data that support the findings of this study are available in the Supporting Information of this article. The fastq sequence data with associated metadata for this study have been deposited at the National Center for Biotechnology Information (NCBI) under BioProject PRJNA119.

## Conflict of interest statement

The authors declare no competing financial interests that are of commercial interest.

## Supplementary data

Supplementary data is available at *Horticulture Research* online.

## Supplementary Material

Web_Material_uhae111
