# Supplementary material for: Enhancing tomato fruit antioxidant potential through hydrogen nanobubble irrigation
Source: Hortic Res. 2024 Apr 16;11(6):uhae111. doi: 10.1093/hr/uhae111 (PMC11186064; doi:10.1093/hr/uhae111)
Supplement: Web_Material_uhae111 [file web_material_uhae111.zip › Supplementary Data-highlight.docx]

***Horticulture Research* Supporting Data**

Article title: Enhancing Tomato Fruit Antioxidant Potential Through Hydrogen Nanobubble Irrigation

Authors: Jing He†, Yunpeng Zhou†, Christoph-Martin Geilfus, Jiankang Cao, Daqi Fu, Shahar Baram, Yanzheng Liu, Yunkai Li**^*^**

Article acceptance date: 14 December 2023

The following Supporting Information is available for this article:

**Fig. S1 Photographs taken during the plant growth and after plant harvest.**

**Fig. S2 The qRT-PCR assay of different expression genes in KEGG pathway.**

**Fig. S3 KEGG pathway enrichment analysis of the various genes in the comparison groups.**

**Fig. S4 The qRT-PCR assay of different expression genes in KEGG pathway.**

**Fig. S5 The expression of NADH dehydrogenase-related genes.**

**Fig. S6 Analysis of the correlation between genes with varying antioxidant properties.**

**Fig. S7 The concentration of ·OH in fruits tissues.**

**Fig. S8 The qRT-PCR assay and measurement of hormones.**

**Fig. S9. HNBs and ONBs promoted the antioxidant capacity of tomatoes.**

**Fig. S10. HNBs and ONBs promoted the yield of tomatoes.**

**Table S1: Irrigation and fertilizer amounts given to tomato plants during the experiments.**

**Table S2: Amount of each reagent used for the ascorbic acid standard curve.**

**Table S3: Amount of each reagent used for the flavonoids standard curve.**

**Table S4: Amount of each reagent used for the glutathione standard curve.**

**Table S5: The primer sequences of qPCR**

**Methods S1 Plant growing conditions**

**Methods S2 AsA analysis**

**Methods S3 LYC analysis**

**Methods S4 Flavonoids analysis**

**Methods S5 GSH analysis**

**Methods S6 Resveratrol analysis**

**Methods S7 Validation of selected DEGs by qRT-PCR**

**Fig. S1 Effect of tomato fruit on treatment groups' transcriptome and metabolome.** Shown are the information for statistical analysis of two omics. Principal component analysis (PCA) of genes (a) and metabolites (d) from three treatments. Circle diagram of GO functional enrichment analysis of differential genes CK vs ONBs (b) and CK vs HNBs (c), respectively. Circle diagram of KEGG functional enrichment analysis of differential genes CK vs ONBs (e) and CK vs HNBs (f), respectively.

**
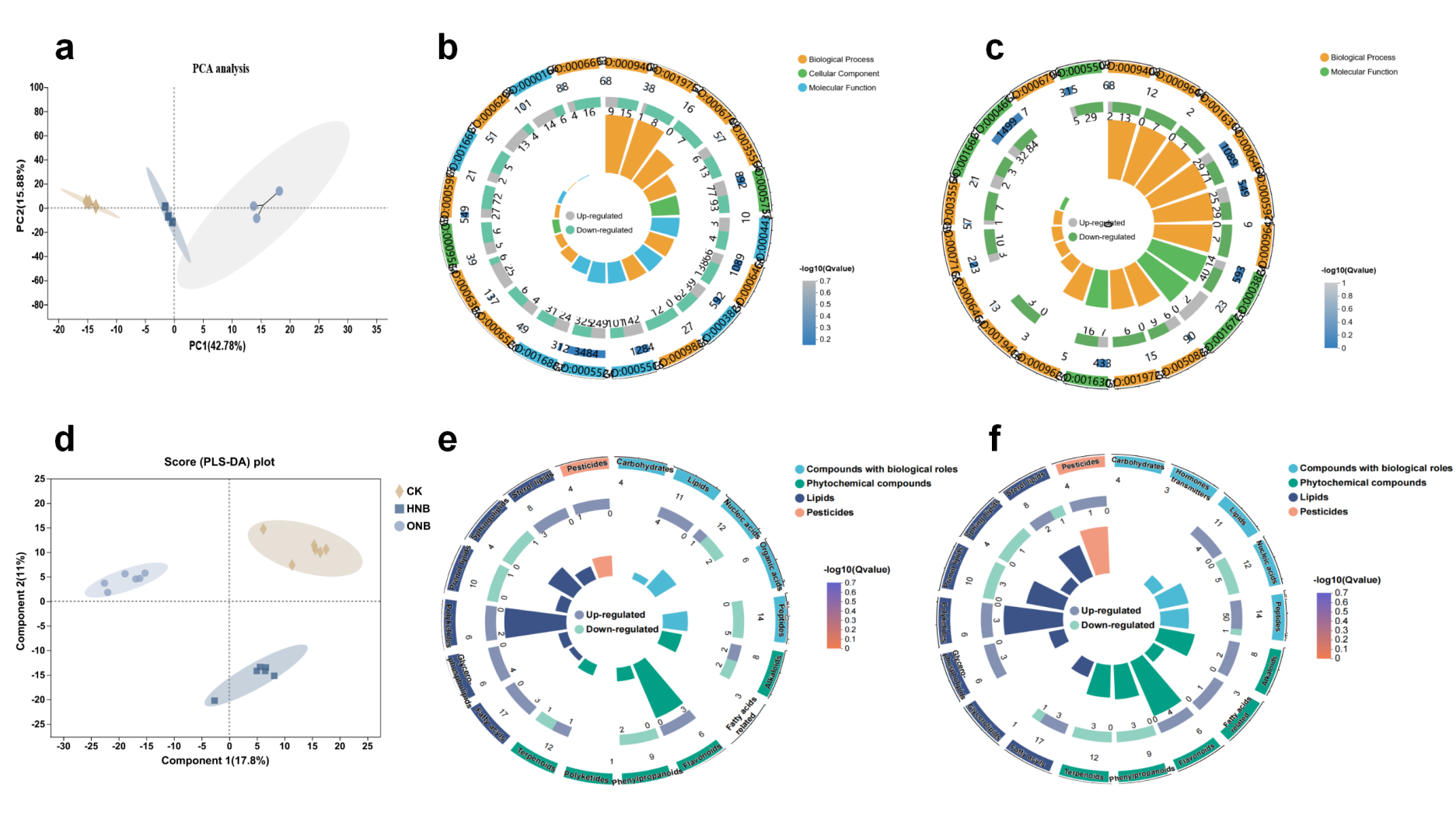
**

**Fig. S2 The qRT-PCR assay of different expression genes in KEGG pathway.** The mRNA relative expression (of CK) for key enzymes MDHAR and GST (a) in ascorbate and aldarate metabolism. *MDHAR* and *GST* were upregulated by 2.4-6.2 and 3.4-4.6 folds compared with CK, respectively. The mRNA relative expression (of CK) for enzymes AAO3, BCH2, CYP707A1 and LUT5 (b) in carotenoid biosynthesis. *AAO3, BCH2, CYP707A1* and *LUT5* were upregulated by 12.6-19.6, 2.5-5.6, 3.8-6.6, and 5.3-6.4 folds compared with CK, respectively. In addition, we report statistical significance of F-tests for the main effect of them, and marked by *, **, *** and **** for p-values < 0.05, < 0.01, < 0.001 and < 0.0001, respectively. Error bars show the standard errors of the means.


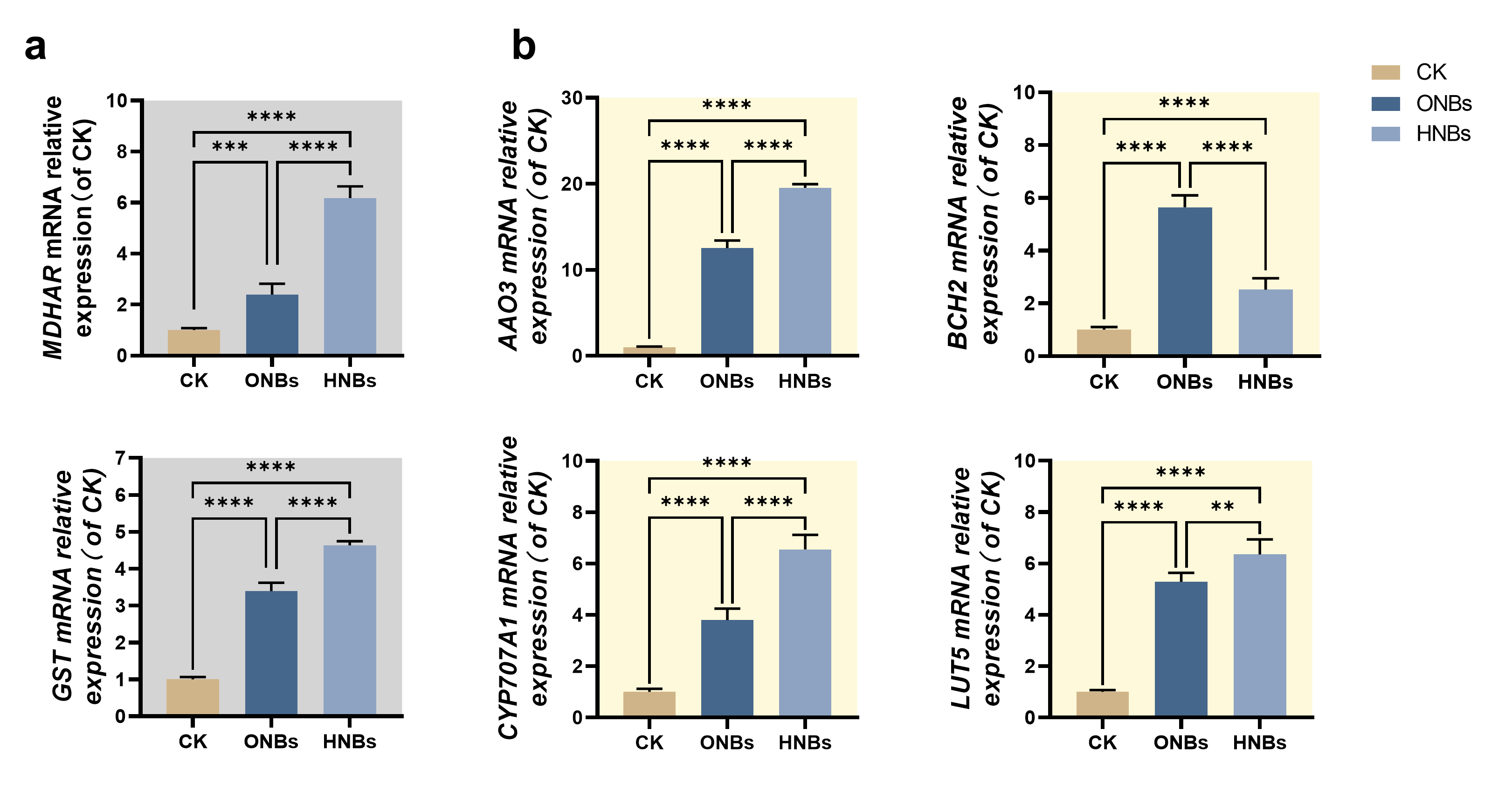


**Fig. S3 KEGG pathway enrichment analysis of the various genes in the comparison groups.** The horizontal axis of the column chart indicates the number of differential genes enriched in the pathway, and the * indicate the significance of differential gene enrichment in the pathway (*, **, and *** for p-values < 0.05, < 0.01, and < 0.001, respectively).

**
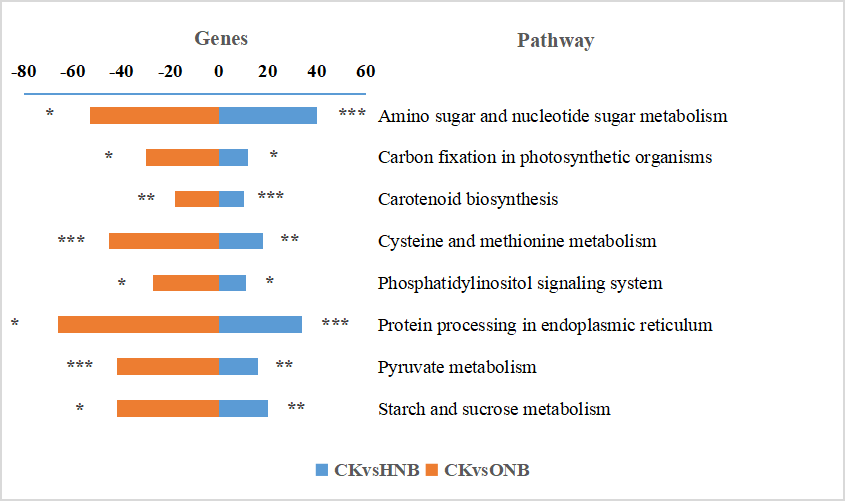
**

**Fig. S4. The qRT-PCR assay of different expression genes in KEGG pathway.** The mRNA relative expression (of CK) for ATP-producing genes (a) were upregulated by 6.8-19.0, 1.7-3.9 and 11.1-44.7 folds compared with CK, respectively. The mRNA relative expression (of CK) for *Cytb* and *COX1* were upregulated by 5.5-11.0, and 8.4-13.3 folds compared with CK, respectively. In addition, we report statistical significance of F-tests for the main effect of them, and marked by *, **, *** and **** for p-values < 0.05, < 0.01, < 0.001 and < 0.0001, respectively. Error bars show the standard errors of the means.

**
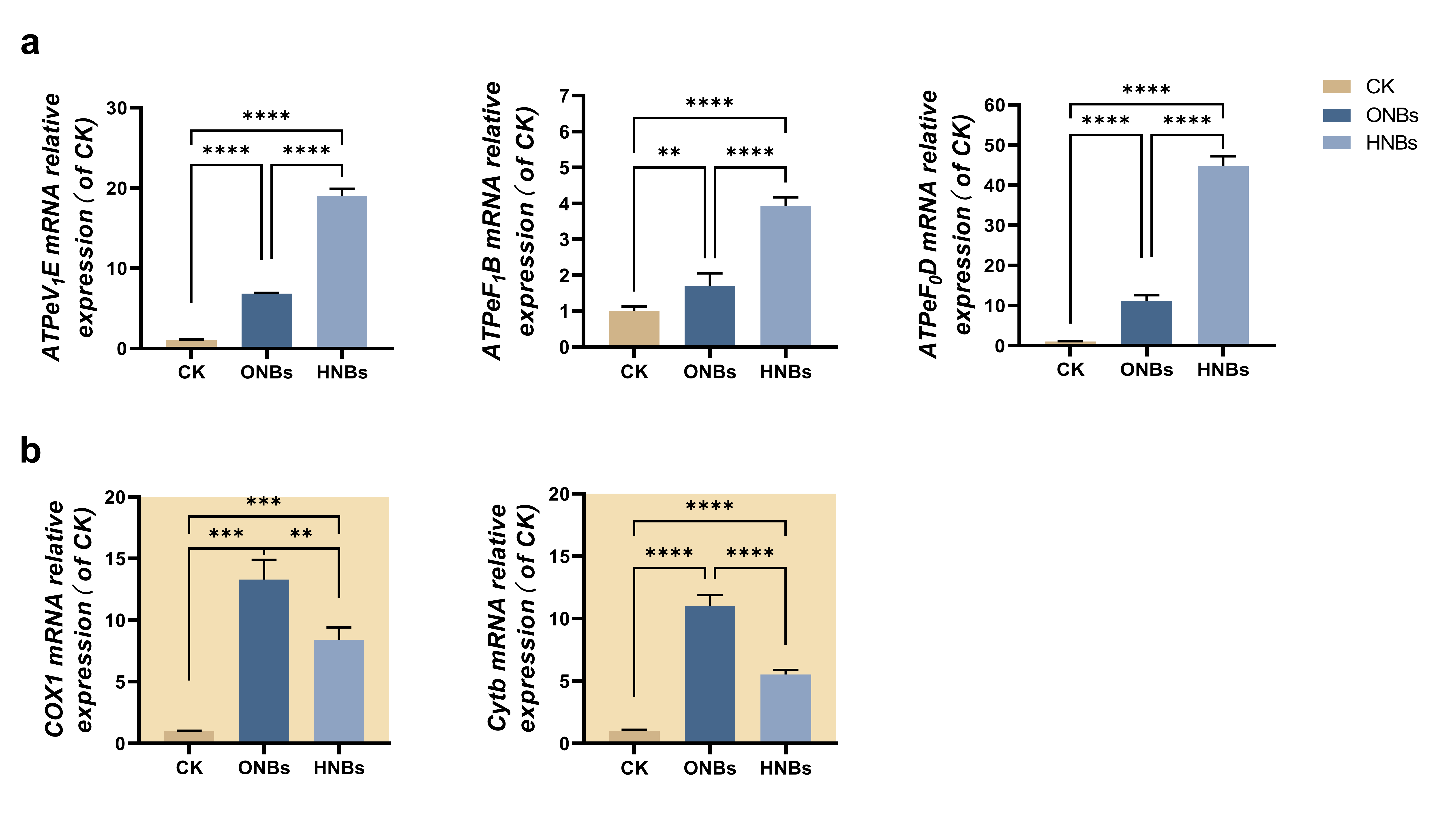
**

**Fig. S5. The expression of NADH dehydrogenase-related genes.** The gene abundance for *NDUFS1* and *NDUFA8* were upregulated by 20.7% and 35.8% compared with ONBs, respectively. Differential gene expression analysis of multiple samples using DESeq2, FC>1.5, and marked by *, **, *** for p-values < 0.05, < 0.01, < 0.001, respectively. Error bars show the standard errors of the means.


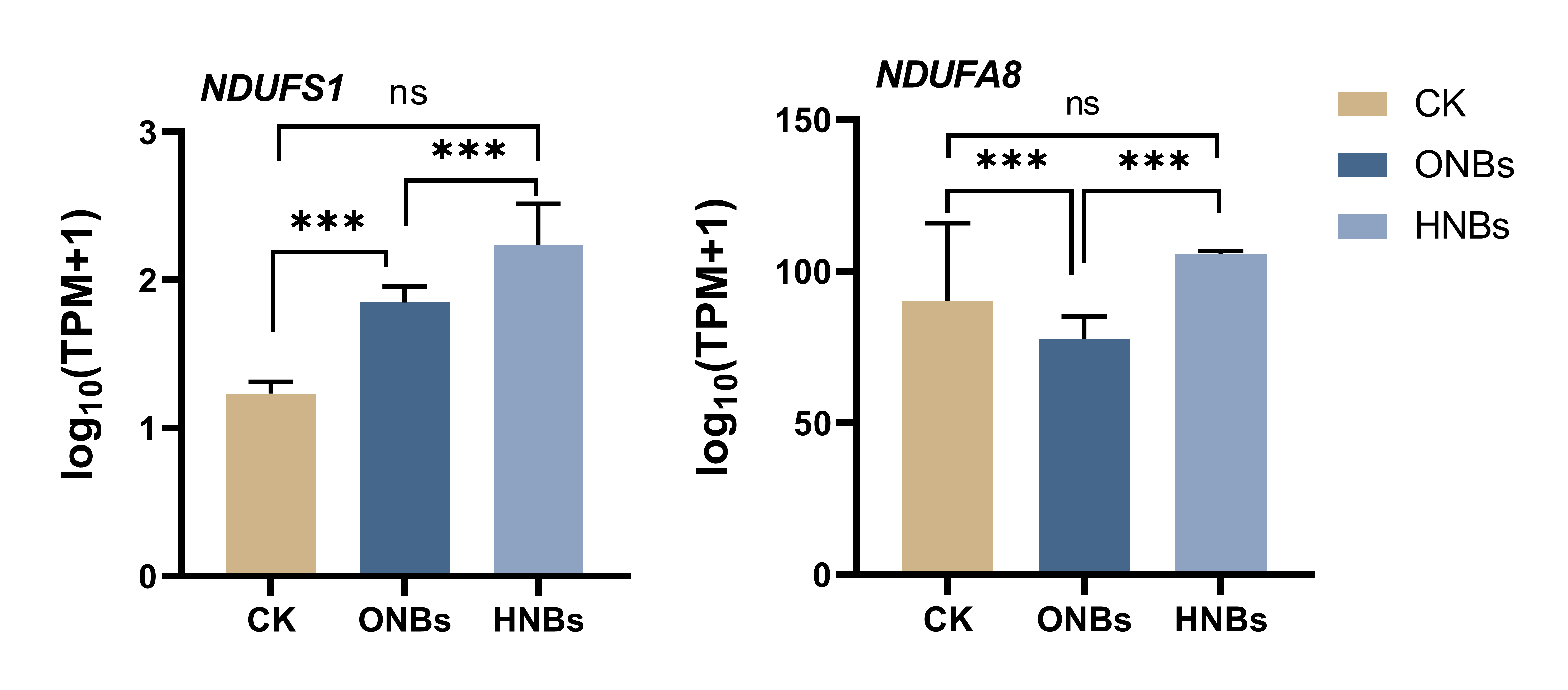


**Fig. S6. Analysis of the correlation between genes with varying antioxidant properties.** Shown correlation information by Pearson of antioxidant biosynthesis-related genes. The color indicates the size of the p-value of the correlation. The larger circle within the square indicates a stronger correlation between the genes.

**
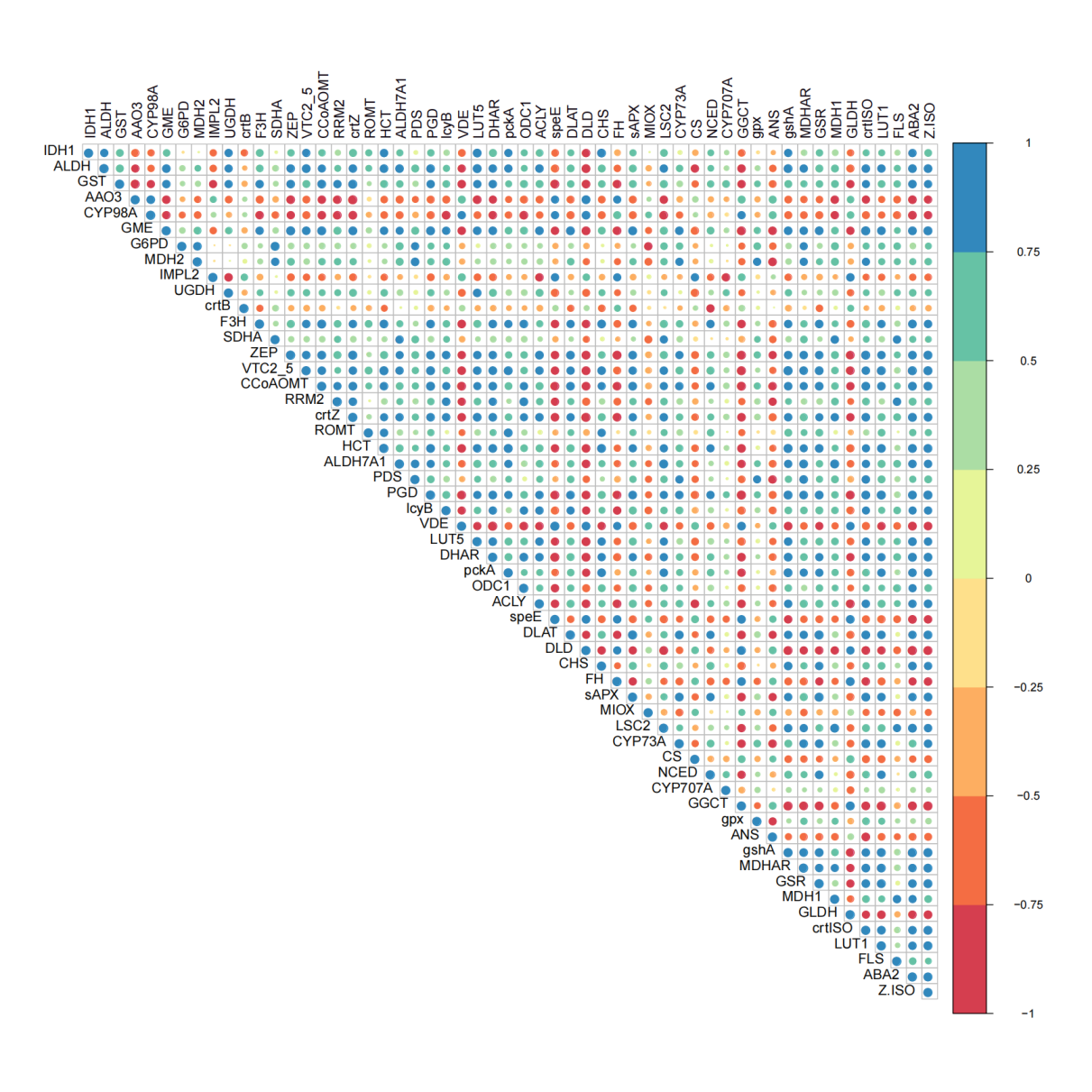
**

**Fig. S7. The concentration of ·OH in fruits tissues.** Nanobubble irrigation increased the ·OH content of plants compared to water irrigation, by 9.0% in the case of HNBs and 35.8% in the case of ONBs. In addition, we report statistical significance of F-tests for the main effect of them, and marked by *, **, *** and **** for p-values < 0.05, < 0.01, < 0.001 and < 0.0001, respectively. Error bars show the standard errors of the means.

**
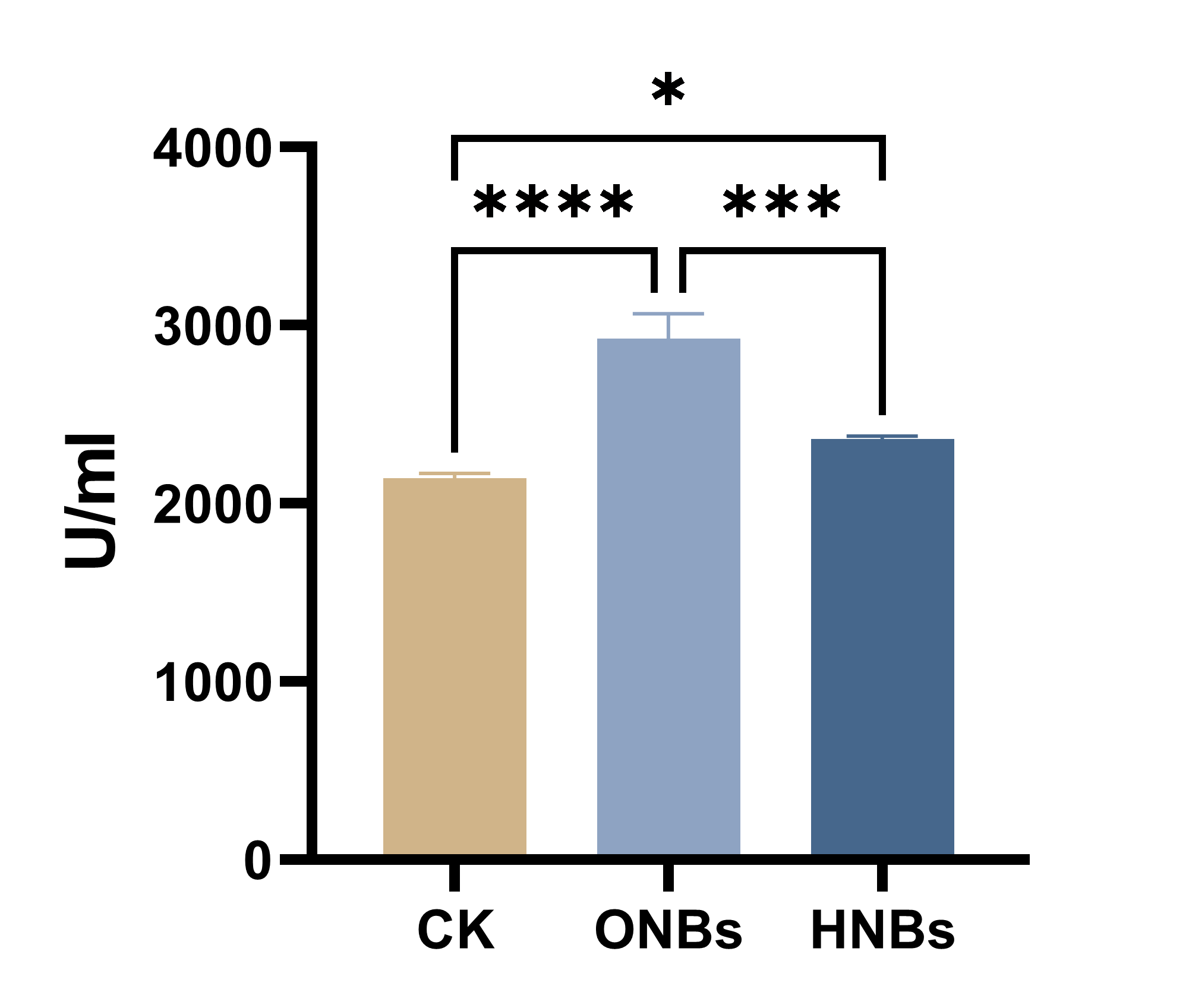
**

**Fig. S8. The qRT-PCR assay and measurement of hormones.** The mRNA relative expression (of CK) for key enzymes BIN2 and ABA2 were upregulated by 4.0-6.3 and 9.6-12.0 folds respectively compared with the CK (a). The concentration of ABA and jasmonic acid were improved by 1.2-1.3 and 1.1-1.4 fold, respectively (b). In addition, we report statistical significance of F-tests for the main effect of them, and marked by *, **, *** and **** for p-values < 0.05, < 0.01, < 0.001 and < 0.0001, respectively. Error bars show the standard errors of the means.


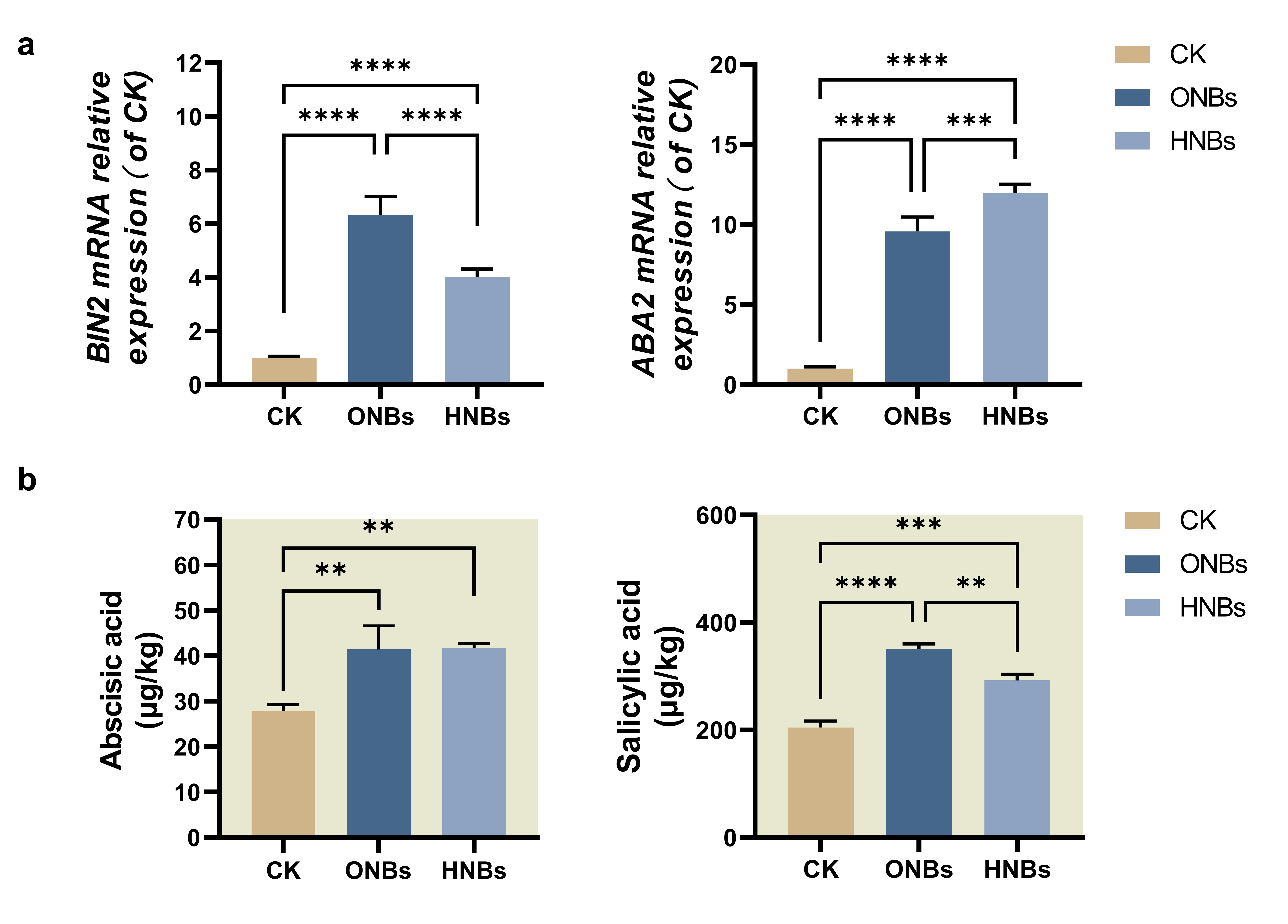


**Fig. S9. HNBs and ONBs promoted the antioxidant capacity of tomatoes.** HNBs increased the antioxidant capacity of tomatoes compared to the CK and ONBs. The increase of enzymes’ activity with SOD, GSH-Px and APX (a) were greater by 17.6%, 12.9%, 18.3% than ONBs, and greater by 37.6%, 50.1 and 64.5% than CK. The radical scavenging capacity by hydroxyl (·OH), 2,2′-azino-bis (3-ethylbenzothiazoline-6-sulfonic acid) (ABTS) and 2,2-diphenyl-1-picrylhydrazyl (·DPPH) were greater by 7.3%, 4.9%, 14.2% than ONBs, and greater by 65.5%, 32.8 and 29.7% than CK. In addition, we report statistical significance of F-tests for the main effect of them, and marked by *, **, *** and **** for p-values < 0.05, < 0.01, < 0.001 and < 0.0001, respectively. Error bars show the standard errors of the means.

**
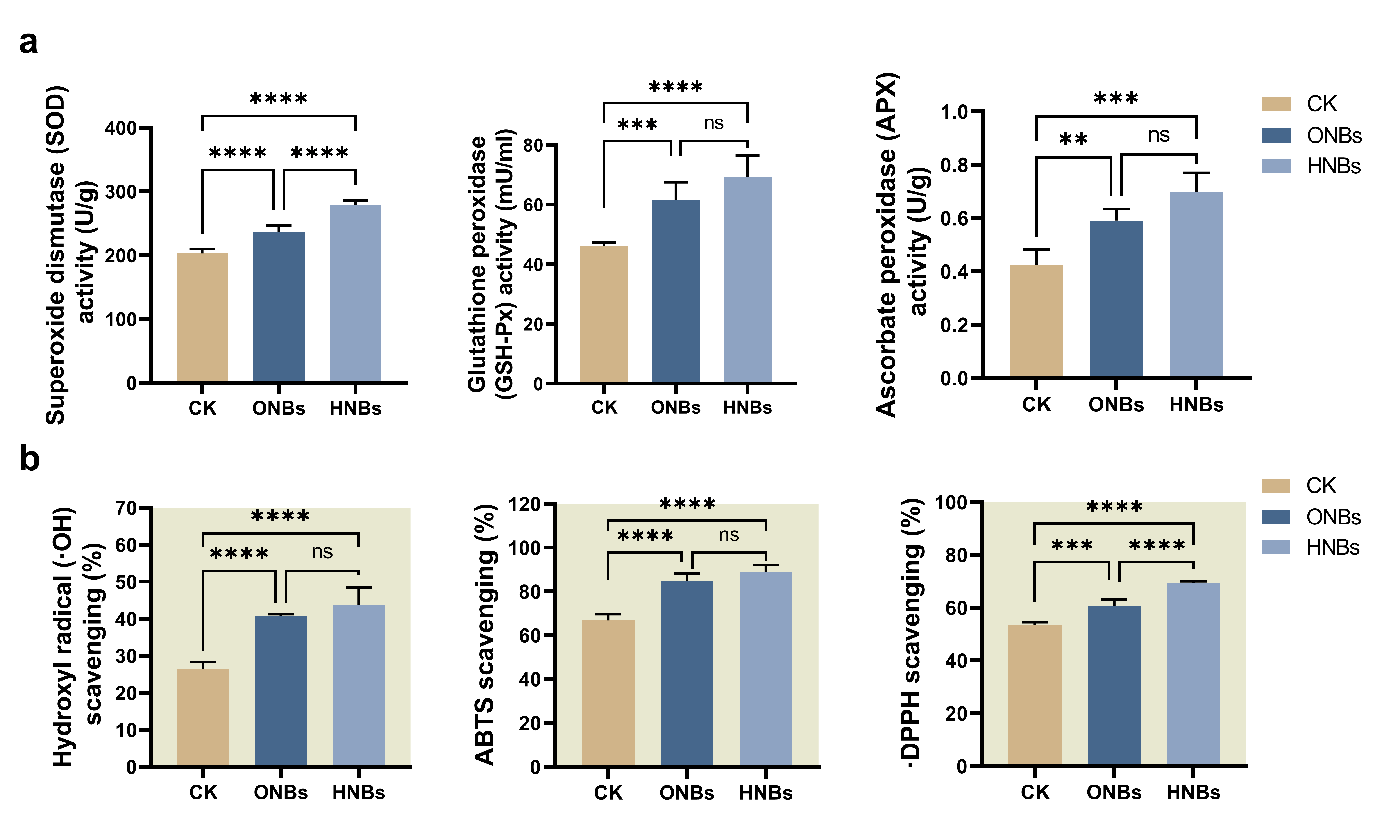
**

**Fig. S10. HNBs and ONBs promoted the yield of tomatoes.** HNBs increased the average yield of tomatoes compared to the CK and ONBs, the increase was greater by 22.5% and 2.4% than CK and ONBs. In addition, we report statistical significance of F-tests for the main effect of them, and marked by *, **, *** and **** for p-values < 0.05, < 0.01, < 0.001 and < 0.0001, respectively. Error bars show the standard errors of the means.


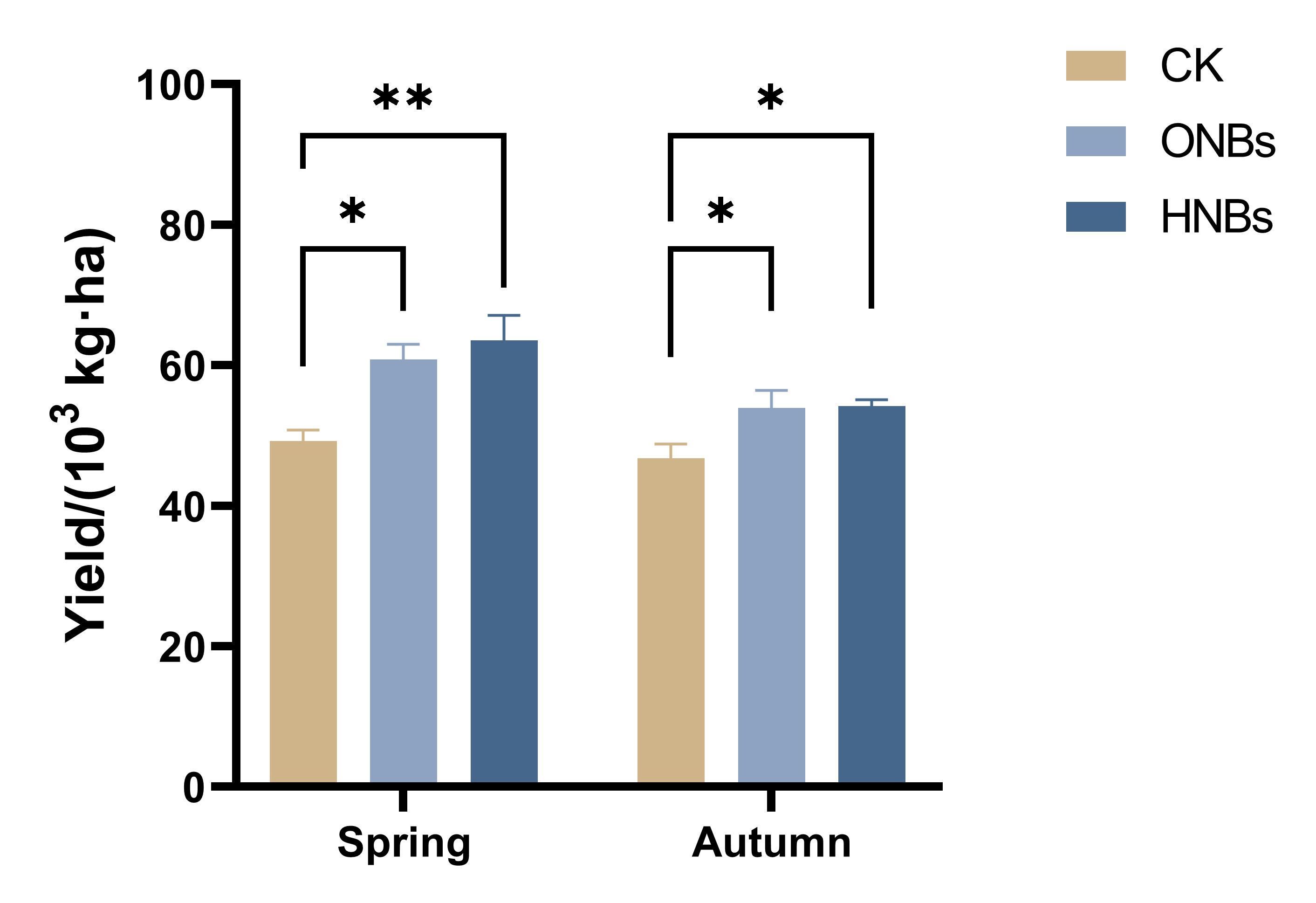


**Methods S1 Plant growing conditions**

For the plant growth experiment, the tomato seedlings were grown in the greenhouse (60 m long and 8 m wide) of the Tongzhou Experimental Station of China Agricultural University (Beijing, China, 116.70 °E, 39.71 °N).

The monthly sunshine duration of Tongzhou Experimental Station of China Agricultural University in spring and autumn was 290 and 245 h, respectively. Rui fen 882 tomato seedlings (Rijk Zwwan, Netherlands) were used to study fruit quality and yield. Under the same environment, the seeds were seeded and cultivated in the plug, and germinated for 35 days before colonization in the experimental area. Spring tomatoes were transplanted on March 26^th^, 2020 and harvested on June 30^th^, 2020. Fall tomatoes were transplanted on July 5^th^, 2020 and harvested on October 12^th^, 2020.

**Table S1 Irrigation and fertilizer amounts given to tomato plants during the experiments.**

| Growth period | Irrigation times | Irrigation volume  (m^3^/ha) | Fertilization  times | Cow manure  (10^3^ kg/ha) | Compound fertilizer  (kg/ha) | CH_4_N_2_O/  (kg/ha) | KH_2_PO_4_/  (kg/ha) | K_2_SO_4_/  (kg/ha) |
| --- | --- | --- | --- | --- | --- | --- | --- | --- |
| Planting | 1 | 300 | 1 | 45 | 750 | 0 | 0 | 0 |
| Seeding stage | 2 | 120 | 4 | 0 | 0 | 19.5 | 2.25 | 9 |
| Flowering and fruit bearing stage | 4 | 150 | 4 | 0 | 0 | 76.5 | 7.5 | 69 |
| Fruit enlarging stage | 4 | 300 | 10 | 0 | 0 | 39 | 0 | 105 |
| Full fruit stage | 7 | 225 |  |  |  |  |  |  |
| Summary | 18 | 3915 | 19 | 45 | 750 | 774 | 84 | 1362 |

Note: Compound fertilizer, N:P_2_O_5_: K_2_O=15: 15: 15; CH_4_N_2_O, urea; KH_2_PO_4_, potassium dihydrogen phosphate; K_2_SO_4_, potassium sulfate. After tomato entering fruit enlarging stage, potassium and calcium fertilizer were foliar sprayed every 7 days, totally 3 times.

**Methods S2 AsA analysis**

Each sample (10 g) was crushed and extracted three times with 5% (w/v) trichloroacetic acid (TCA). The extracts were transferred to a 100 mL volumetric flask, and TCA was added to the scale. The supernatant was obtained by centrifugation at 4 °C, 12000 rpm. Then, 1 mL of the supernatant was mixed with 1 mL TCA, 1 mL anhydrous ethanol, 0.5 mL phosphoric acid-ethanol (0.4%), 1 mL bathophenanthroline-ethanol (5 g/L) and 0.5 mL FeCl_3_-ethanol (0.3 g/L). The mixture was incubated at 30 °C for 60 min.

**Table S2** Amount of each reagent used for the ascorbic acid standard curve.

| Reagent name | Concentration | Number | | | | | | |
| --- | --- | --- | --- | --- | --- | --- | --- | --- |
|  |  | 1 | 2 | 3 | 4 | 5 | 6 | 7 |
| ascorbic acid standard solution (mL) | 100μg/mL | 0 | 0.1 | 0.2 | 0.3 | 0.4 | 0.5 | 0.6 |
| TCA (mL) | 50g/L | 2.0 | 1.9 | 1.8 | 1.7 | 1.6 | 1.5 | 1.4 |
| Anhydrous ethanol (mL) | - | 1.0 | 1.0 | 1.0 | 1.0 | 1.0 | 1.0 | 1.0 |
| PA-EtOH (mL) | 0.4% | 0.5 | 0.5 | 0.5 | 0.5 | 0.5 | 0.5 | 0.5 |
| BP-EtOH (mL) | 5g/l | 1.0 | 1.0 | 1.0 | 1.0 | 1.0 | 1.0 | 1.0 |
| FeCl_3_-EtOH (mL) | 0.3g/L | 0.5 | 0.5 | 0.5 | 0.5 | 0.5 | 0.5 | 0.5 |
| Analogous ascorbic acid content (μg) | - | 0 | 10 | 20 | 30 | 40 | 50 | 60 |

Note: TCA, trichloroacetic acid; PA-EtOH, a mixture of phosphoric acid and anhydrous ethanol; BP-EtOH mixture of 4,7-diphenyl-1,10-phenanthroline and anhydrous ethanol; FeCl_3_-EtOH, a mixture of ferric chloride and anhydrous ethanol. Test tube No. 1 was used as the reference. The standard curve was plotted using the analogous ascorbic acid content as the horizontal coordinate and the absorbance value as the vertical coordinate.

**Methods S3 LYC analysis**

One gram of homogenized sample was mixed with 0.1% butylated hydroxytoluene -ethanol in a brown centrifuge tube. LYC was extracted from the mixture with an oscillator under 25 °C for 4 h. The non-polar phase was filtered through a 0.22 µm filter membrane (Sigma-Aldrich, St. Louis, Missouri, U.S.A) and injected into the brown sample bottle. Compounds were separated by HPLC1200 system fitted with a C18 reverse phase HPLC column (0.5 µm, 250 × 4.6 mm). The test was carried out under the following conditions: oven temperature, 40 °C; flow rate, 1.0 mL/min; mobile phase, methanol and acetonitrile (35 : 65).

**Methods S4 Flavonoids analysis**

**Table S3** Amount of each reagent used for the flavonoids standard curve.

| Reagent name | Concentration | Number | | | | | |
| --- | --- | --- | --- | --- | --- | --- | --- |
|  |  | 1 | 2 | 3 | 4 | 5 | 6 |
| Rutin standard solution (mg/mL) | 100μg/mL | 0.5 | 1 | 2 | 3 | 4 | 5 |
| HCl-MeOH (mL) | - | 19.5 | 19 | 18 | 17 | 16 | 15 |
| Analogous flavonoids content (μg/mL) | - | 2.5 | 5 | 10 | 15 | 20 | 25 |

Note: HCl-MeOH, mixture of HCl (1mL) and methyl alcohol (99mL). Test tube No. 1 was used as the reference. The standard curve was plotted using the analogous flavonoids content as the horizontal coordinate and the absorbance value as the vertical coordinate.

**Methods S5 GSH analysis**

Five grams of the sample were ground in a mortar with 5 mL of a pre-cooled 50 g/L TCA solution containing ethylenediaminetetraacetic acid disodium salt. The homogenate was centrifuged at 12000 rpm at 4 °C for 20 min. The supernatant (1 mL) was mixed with 1 mL phosphate buffer (PBS, 0.1 mol/L, pH 7.7) as the No.1 solution. The same conditions were used to set the No.2 solution [4 mmol/L 5,5'-Dithiobis-(2-nitrobenzoic acid), 0.5 mL] and PBS (0.5 mL 0.1 mol/L at pH 6.8). The absorbance of each solution was then measured at 412 nm. The glutathione (GSH) content was determined by estimating the difference in absorbance between the two solutions and calculated by comparison with authentic GSH (y = 0.1209x; R^2^ = 0.9821) ([Sahoo](#_ENREF_34) *[et al.](#_ENREF_34)*[, 2017](#_ENREF_34)).

**Table S4** Amount of each reagent used for the glutathione standard curve.

| Reagent  name | Reagent concentration | Number | | | | | |
| --- | --- | --- | --- | --- | --- | --- | --- |
|  |  | 1 | 2 | 3 | 4 | 5 | 6 |
| Reduced glutathione standard solution (mL) | 100μmol/L | 0 | 0.2 | 0.4 | 0.6 | 0.8 | 1.0 |
| Distilled water (mL) | - | 1.0 | 0.8 | 0.6 | 0.4 | 0.2 | 0 |
| PBS (pH 7.7) (mL) | 0.1μmol/L | 1.0 | 1.0 | 1.0 | 1.0 | 1.0 | 1.0 |
| DTNB (mL) | 4mmol/L | 0.5 | 0.5 | 0.5 | 0.5 | 0.5 | 0.5 |
| Analogous glutathione content (μmoL/g) | - | 0 | 20 | 40 | 60 | 80 | 100 |

Note: PBS, phosphate buffer solution; DTNB, mixture of 5,5-dithiobis-2-nitrobenzoic acid (15.8 mg) and PBS (pH 6.8, 0.1 mol/L) Test tube No. 1 was used as the reference. The standard curve was plotted using the analogous glutathione content as the horizontal coordinate and the amount of the reduced glutathione material as the vertical axis.

**Methods S6 Resveratrol analysis**

One gram of the sample was placed in a centrifuge tube, and 5 mL methanol was added after 30 min of sonication. The mixture was centrifuged twice at 4500 rpm for 10 min, and the supernatants were pooled and filtered using a 0.22 µm filter membrane for further use. Compounds were separated by HPLC1200 system fitted with a C18 reverse phase HPLC column (0.5 µm, 250 × 4.6 mm). The test was carried out under the following conditions: oven temperature, 40 °C; flow rate, 1.0 mL/min; mobile phase, acetonitrile and water.

**Methods S7 Validation of selected DEGs by qRT-PCR.**

The PCR amplification was carried out using the following conditions: 95 °C for 10 min, followed by 40 cycles at 95 °C for 15 s, and 60 °C for 30 s. The relative expression was evaluated using the delta-delta Ct (2^−△△Ct^) method based on three biological replicates.

**Table S5 The primer sequences of qPCR**

|  | Prime | Sequence (5ʼ-3ʼ) |  | Prime | Sequence (5ʼ-3ʼ) |
| --- | --- | --- | --- | --- | --- |
| GAPDH | GAPDH-F | 5'-AGGTCGGTGTGAACGGATTTG -3' | COX1 | COX1-F | 5'-GCGACTCGGAGGACATAAGA-3' |
|  | GAPDH-R | 5'-GGGGTCGTTGATGGCAACA-3' |  | COX1-R | 5'-CACAGTTCACTCGCGTTGAT-3' |
| BIN2 | BIN2-F | 5'-GCAGAGGAGAAGAGAGCACA-3' | NADH | NADH-F | 5'-GGAGGAGAGAGACAGCTTCC-3' |
|  | BIN2-R | 5'-GGATTCCATTTCCGGTCGTG-3' |  | NADH-R | 5'-AGATTCCCCAGCTGCACTAA-3' |
| ABA2 | ABA2-F | 5'-GTGGAGATGAGTTGAGGGGT-3' | DHAR | DHAR-F | 5'-ACAGGACCTCTCAGATGCAA-3' |
|  | ABA2-R | 5'-CAACAGAGGGAAGGTCGAGT-3' |  | DHAR-R | 5'-AAGTGCTACTCCCTTCGTGG-3' |
| ETR | ETR-F | 5'-TCTGGTAGAGATGAGGCCCT-3' | lcyB/crtZ | CrtZ-F | 5'-CCAGTCCCCTCCATGCTTAA-3' |
|  | ETR-R | 5'-TTCCCACCATCAGCTCCAAT-3' |  | CrtZ-R | 5'-AGGCGGATTTTGGACTGAGA-3' |
| ATPeV1E | ATPeV1E-F | 5'-CGATGTGTGTTGCTGGCATA-3' | AAO3 | AAO3-F | 5'-CTGGAAGTCATGGGGTCAGT-3' |
|  | ATPeV1E-R | 5'-CTGCATCGTTCATCTTCGCA-3' |  | AAO3-R | 5'-GCAAAGTAAGGAGCGAGTGG-3' |
| ATPeF1B | ATPeF1B-F | 5'-CTTATGGCCTCTCTCCTCCG-3' | CYP707A1 | CYP707A1-F | 5'-CAGGTGGGAAGTGGTAGGAT-3' |
|  | ATPeF1B-R | 5'-ACTTAGGAGGTGTCGCTGAC-3' |  | CYP707A1-R | 5'-GGGGTTTGGGGTTGATAAGC-3' |
| ATPeF0D | ATPeF0D-F | 5'-AGCACATCAAACAGACAGCC-3' | LUT5 | LUT5-F | 5'-TCAACGGGTCGATTCTTCCA-3' |
|  | ATPeF0D-R | 5'-TCGGTGACAAGAAGCTTTGC-3' |  | LUT5-R | 5'-GGGGAGGTGGCTTATGAGTT-3' |
| Cytb | Cytb-F | 5'-CCTCCAGAACCCCGTAAGTT-3' |  |  |  |
|  | Cytb-R | 5'-CTCTGGAAAATTGGGAGCGG-3' |  |  |  |
